# Incisal‐Third Tunnel Splint (ITTS) Technique for Interim Stabilization of Mobile Anterior Teeth: A Two‐Case Report

**DOI:** 10.1155/crid/1381109

**Published:** 2026-09-30

**Authors:** Fahad Hussain Al-Qahtani

**Affiliations:** ^1^ Consultant in Advanced Restorative and Esthetic Dentistry Center for Advanced Consultants in Healthcare Education and Training (CACHET) Riyadh Kingdom of Saudi Arabia

**Keywords:** anterior teeth, incisal-third preparation, interim stabilization, periodontal mobility, staged rehabilitation, tooth splinting

## Abstract

Full‐mouth extraction and replacement are usually staged, and the sequence may leave mobile anterior teeth in service for months before their planned removal—an interval calling for stabilization that is quick, well‐tolerated, and appropriate for teeth shortly to be extracted. Two such intervals were managed with an incisal‐third tunnel splint (ITTS): a shallow tunnel cut within the incisal third of the mandibular anterior teeth, receiving a passive stainless‐steel wire enclosed with composite resin. Siting it there rather than lingually places the restoration away from the gingival third and interdental region. Intracoronal splinting is not new; descriptions from the 1970s to the early 1990s concerned teeth intended for retention, where loss of tooth structure told against it. In Case 1, a 75‐year‐old man had fractured, mobile maxillary prostheses on carious abutments and a partially edentulous mandible with advanced bone loss and Grade II–III mobility of the anterior teeth. Staged rehabilitation was planned, beginning with the maxilla, the form of replacement left open; the ITTS was placed on the mandibular anterior teeth to carry them through that interval. At 5.5 months, the maxillary phase had not begun, and the splint remained in service. In Case 2, a 64‐year‐old woman underwent posterior‐to‐anterior mandibular implant rehabilitation; the splint served approximately 1 year and was removed at anterior implant placement as planned. Both remained intact and bonded, with no discomfort, phonetic disturbance, or hygiene difficulty reported. No mobility index, periodontal charting, or vitality testing was performed, and no comparative data are presented. The technique was observationally feasible and well‐tolerated; generalizable utility cannot be inferred from two uncontrolled cases, and it is offered as one splinting option for this interval rather than a preferred method.

## 1. Introduction

Advanced attachment loss makes itself felt: The teeth move, chewing feels insecure or painful, and the patient senses that the teeth are failing; anteriorly, drifting and spacing add an unwelcome change in appearance [1, 2]. Where remaining support is inadequate, extraction is the appropriate definitive treatment. What follows it is a separate decision: implant‐supported replacement [3–5]; a removable prosthesis, where cost, general health, or the burden of advanced surgery makes implant treatment unsuitable; or, in some patients, no prosthetic replacement at all.

Such treatment may be completed in one episode: Where bone, primary stability, and the patient′s general condition allow, the remaining teeth are cleared, implants placed immediately, and a provisional prosthesis delivered the same day—and no interval arises. Where that route is unsuitable or not chosen, the work is staged, and the sequence governs how long the teeth awaiting extraction stay in the mouth: Where both arches are involved, one may be treated ahead of the other, usually that with the greater loss of support [18]; where the problem is confined to a single arch, treatment proceeds within it from posterior to anterior. Either way, an interval of several months arises in which mobile anterior teeth must stay functional and acceptable in appearance without themselves becoming the object of periodontal treatment—routine in staged practice, yet little described.

Splinting mobile teeth into a rigid segment has an established place in periodontal practice, palliative rather than curative [6, 7]; a recent systematic review found no survival benefit in advanced periodontitis while noting its continued use for patient comfort [8]. Here, that is no shortcoming: The teeth are agreed for extraction, and comfort and function through the interval are all that is asked.

Fixed splints are usually bonded to the lingual surfaces. Contemporary practice favors fiber‐reinforced composite (FRC), which needs no tooth preparation, looks better than a metal element, and is the usual reference approach for provisional stabilization [9, 10]; reported performance is variable, with debonding and delamination predominating among failures [9]. Lingual placement of any kind carries practical costs: It lies where cleaning is difficult and support already compromised, contacts the tongue in speech and swallowing, and is loaded in a manner that invites debonding [1, 6, 11].

This report describes a variation in which the retentive element is carried within a shallow tunnel preparation in the incisal third rather than bonded to the lingual surface—the incisal‐third tunnel splint (ITTS), which places the restoration away from the gingival margin and the interdental col, and away from the lingual surface [1, 6], and leaves the pre‐existing occlusal contacts unchanged.

The name is used descriptively. Intracoronal, the term of the earlier literature, records only that a preparation lies within the clinical crown; the present term adds what that leaves open—the site, the incisal third, and the form, a tunnel—and with them the limit within which the preparation is confined.

Bonding a wire across the anterior teeth with composite is long established, and retention within the tooth itself is not new either. Intracoronal splinting, in which a preparation is cut within the clinical crown to receive a wire or ligature which is then sealed with amalgam, self‐curing acrylic, or composite resin, was described repeatedly between the 1970s and the early 1990s—at the level of the contact areas, at the occlusal third, and at the incisal edge itself [12–14]. Those accounts predate contemporary bonding agents: Retention was obtained from etched enamel alone or, in the most recent of them, mechanically without etching [14].

Against that background, the preparation described here is modest—a tunnel of the dimensions of a small restoration, cut in teeth already scheduled for extraction—and the materials, stainless‐steel wire, conventional adhesive, and composite resin, are held in any general practice. It is not a treatment for periodontal disease; its relation to earlier intracoronal techniques and to orthodontic retention is set out in Section 5.2.

## 2. Case Presentations

### 2.1. Case 1—75‐Year‐Old Male; Interarch Staged Rehabilitation

A 75‐year‐old man attended complaining of loose teeth, with difficulty and apprehension when biting. Examination showed generalized periodontal breakdown. The maxillary fixed prostheses were fractured and mobile, their abutments carious at root level; the mandible was partially edentulous posteriorly, and the anterior teeth showed Grade II–III mobility, with gingival recession and incisal attrition. There was no acute infection at presentation.

Panoramic examination (Figure 1A) showed advanced generalized alveolar bone loss, with multiple periapical radiolucencies in the maxilla. At the mandibular anterior teeth, there was advanced bone loss, with previous endodontic treatment of the central incisors. According to the patient, this treatment had been performed at another facility approximately 3 months earlier; no records were available. The incisors were asymptomatic, with no clinical sign of acute endodontic pathology.

**Figure 1:**
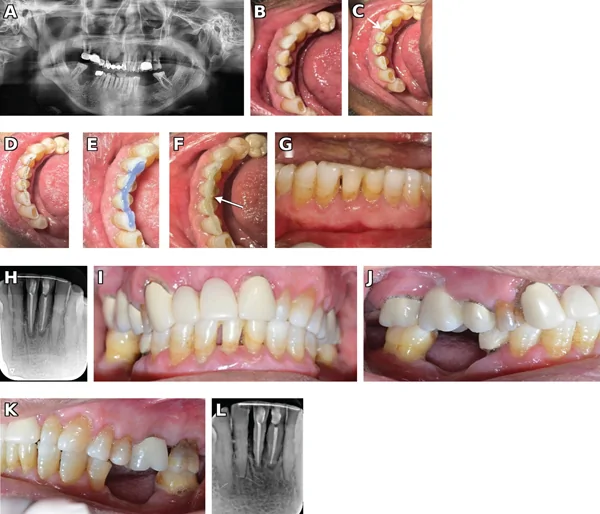
Case 1—75‐year‐old male; interarch staged rehabilitation. (A) Preoperative panoramic radiograph. (B) Preoperative clinical view of the mandibular anterior teeth. (C) Incisal‐third tunnel preparation with a #1/4 round diamond bur under continuous water irrigation; arrow indicates the extent of the tunnel. (D) Trial seating of the 0.7‐mm stainless‐steel wire to verify passive fit before adhesive procedures. (E) Selective phosphoric acid etching of the prepared surfaces. (F) Completed restoration, occlusal view; arrow indicates the composite enclosure. (G) Completed restoration, labial view. (H) Postoperative periapical radiograph showing the position of the wire within the incisal third. (I) Frontal view in centric occlusion at 5.5 months, splint in situ. (J) Right lateral view in centric occlusion at 5.5 months. (K) Left lateral view in centric occlusion at 5.5 months. (L) Periapical radiograph of the mandibular anterior region at 5.5 months, showing the wire, the root canal fillings of the central incisors, the residual pulp space of the remaining splinted teeth, and the level of the surrounding alveolar bone.

On these findings, the mandibular anterior teeth were of poor long‐term prognosis. Full‐mouth extraction was agreed as the definitive plan to be delivered in stages beginning with the maxilla as the source of the periapical lesions. Replacement—implant‐supported or a complete removable prosthesis—was discussed, the choice left open. No periodontal therapy was planned or provided, and retention of these teeth was not an aim of treatment.

The maxillary phase cannot be entered by halves: Once those teeth are removed, the patient is without an upper dentition, so it was timed to his readiness to proceed. Meanwhile, the mandibular anterior segment was stabilized with the ITTS, using a 0.7‐mm stainless‐steel wire per Section 3 (Figure 1B–H). The segment was firm to digital pressure on completion, and the patient reported that biting felt more secure.

The patient was reviewed 5.5 months after splint placement to reassess the treatment plan; the maxillary phase had not yet begun. The splint was intact and fully bonded, with no debonding, wire fracture, or composite fracture, and remained clear of occlusal contact. The patient maintained hygiene without difficulty and reported no discomfort, phonetic disturbance, or awareness of the restoration. Views at this review are shown in frontal and bilateral lateral aspect in centric occlusion (Figure 1I–K) and on a periapical radiograph (Figure 1L). No mobility index, periodontal charting, plaque or bleeding index, or vitality testing was performed at any point.

### 2.2. Case 2—64‐Year‐Old Female; Intra‐Arch Staged Rehabilitation

A 64‐year‐old woman attended with difficulty chewing following the loss of her mandibular molars; mastication was carried on the premolars and the anterior teeth, and she reported the anterior teeth becoming increasingly mobile. She was aware of the state of her dentition and willing to proceed with treatment.

Clinical and radiographic examination showed absent mandibular molars on both sides, the premolars sound and firm, and mandibular anterior teeth with advanced bone loss, Grade II–III mobility, recession, and marked incisal attrition (Figure 2A). Oral hygiene was poor, with no acute swelling or suppuration at presentation.

**Figure 2:**
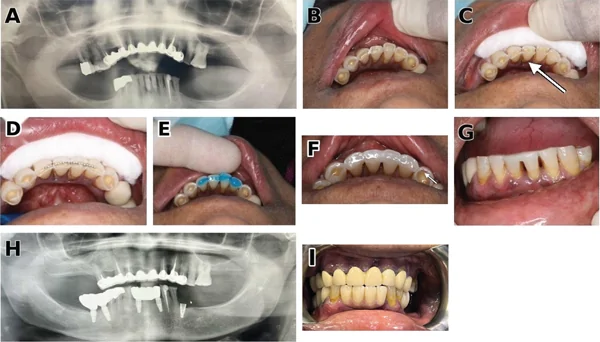
Case 2—64‐year‐old female; intra‐arch staged rehabilitation. (A) Preoperative panoramic radiograph showing absent mandibular molars on both sides and advanced alveolar bone loss at the mandibular anterior teeth. (B) Preoperative clinical view of the mandibular anterior teeth. (C) Field isolation before the splinting procedure; arrow indicates the intended preparation site. (D) Trial seating of the 0.5‐mm stainless‐steel wire to verify passive fit. (E) Selective acid etching at the incisal third. (F) Completed restoration, lingual view; arrow indicates an articulating‐paper marking on the adjacent tooth, with none on the splinted segment. (G) Completed restoration, labial view. (H) Panoramic radiograph at approximately 1 year, following removal of the splint at anterior implant placement. (I) Frontal clinical view in occlusion at approximately 1 year.

Implant‐supported rehabilitation of the mandible was planned as the definitive treatment, proceeding posterior to anterior: Implants placed and restored in the right and left posterior segments first, the anterior teeth extracted and replaced once posterior support was re‐established. As in Case 1, no periodontal therapy was planned or delivered. Stabilization of the anterior segment was required for the posterior phase, and the ITTS was placed with a 0.5‐mm stainless‐steel wire (Figure 2B–F). The segment was firm on completion, and the patient reported improved comfort in function.

Posterior implant placement and restoration proceeded over the following months. The splint remained in service throughout and was removed at approximately 1 year, when anterior implant placement was undertaken, having served its intended purpose. A panoramic radiograph and a frontal clinical view taken at that stage record the completion of the treatment stages the splint had served (Figure 2H,I). The patient reported no discomfort attributable to the splint at any point. The posterior implant restorations were conformed to the occlusal level maintained by the premolars and anterior teeth; the occlusal vertical dimension and pre‐existing contacts were unchanged, whereas the distribution of functional load shifted as posterior support was restored. No periodontal or pulpal records were obtained at any stage, from placement to removal.

## 3. Technical Procedure

The wire diameters differed between the two cases, and the resulting restorations were not mechanically equivalent; they are not replicates of a single protocol.

### 3.1. Step 1—Assessment and Case Selection

The technique is confined to teeth already agreed for extraction: No periodontal treatment is planned for them, and there is no acute infection. Mobility and the state of the incisal edges are examined, and a periapical or panoramic radiograph obtained (Figures 1A and 2A).

### 3.2. Step 2—Tunnel Preparation

A shallow tunnel is cut along the incisal third with a #1/4 round diamond bur (ISO size 005; nominal head diameter 0.5 mm) in a high‐speed handpiece under continuous water irrigation with light intermittent contact, its width matched to the wire. In vital teeth, depth is held to a maximum of 2 mm. In endodontically treated teeth, a slightly deeper preparation may be made if warranted (Figures 1C and 3).

**Figure 3:**
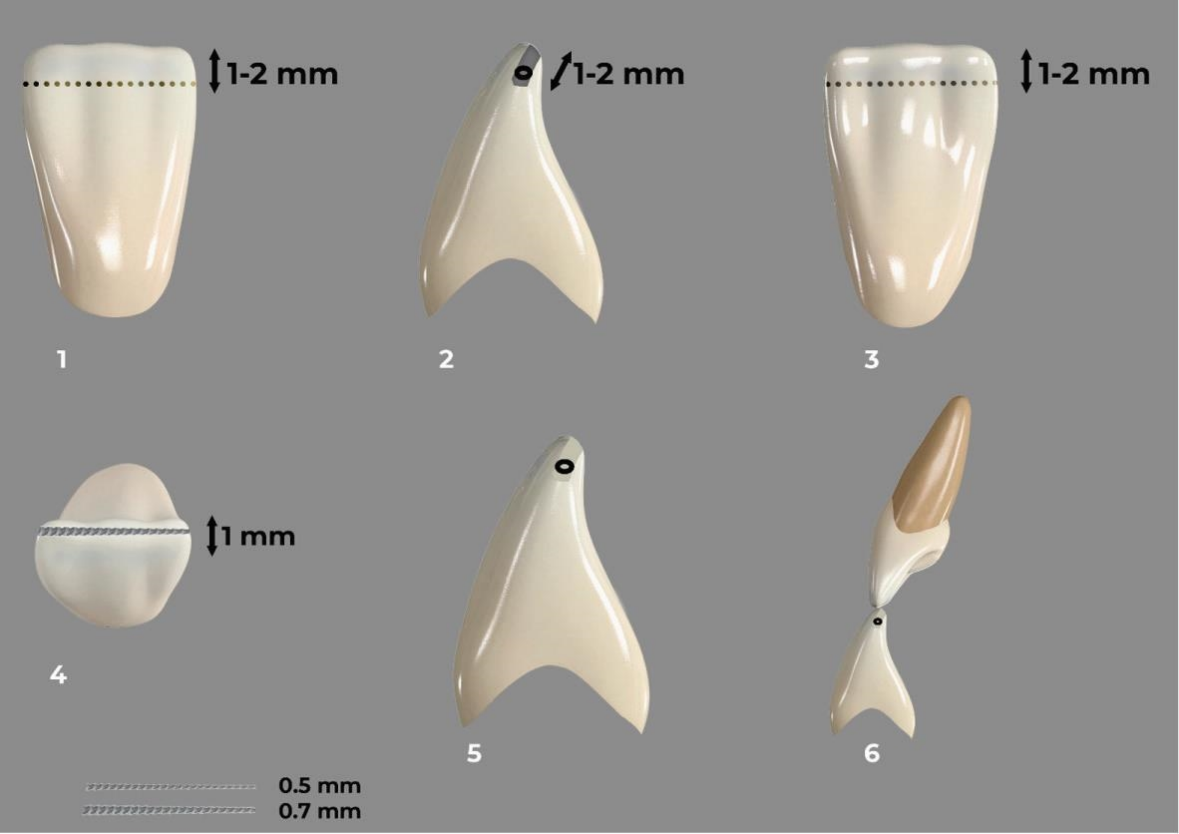
Schematic illustration of the incisal‐third tunnel splint (ITTS) on a mandibular incisor (not to scale). (1) Labial view: The tunnel is prepared within the incisal third at a depth of 1–2 mm from the incisal edge. (2) Proximal view: cross‐section of the wire within the incisal third, 1–2 mm from the incisal edge. (3) Lingual view of the same preparation level. (4) Incisal view: the passive stainless‐steel wire seated within the prepared tunnel; the annotated dimension (1 mm) indicates the tunnel envelope accommodating the wire and its composite cover. (5) Proximal view with the wire fully seated within the tooth body. (6) Occlusal relationship with the opposing maxillary incisor after composite enclosure: The composite is finished to the pre‐preparation height and contour of the incisal edge, so existing occlusal contacts are unchanged even where the opposing tooth meets the composite—the principle applied in Class VI restorations of the incisal edge. Wire diameters used: 0.5 mm (Case 2) and 0.7 mm (Case 1).

### 3.3. Step 3—Wire Selection and Trial Seating

A stainless‐steel wire is selected according to the width of the tunnel, and kept slightly undersized so that it seats without tension, fully enclosed within the incisal edge, as in the 1993 report of Baar et al. [14]. A 0.7‐mm wire was used in Case 1, whereas a 0.5‐mm wire was used in Case 2. It is trial‐seated before any adhesive procedure and adjusted until fully seated within the tunnel, entirely passive and clear of opposing contact (Figures 1D and 2D).

### 3.4. Step 4—Adhesive Conditioning

The prepared surfaces are selectively etched with phosphoric acid, rinsed and gently dried, and a two‐step etch‐and‐rinse adhesive (Adper Single Bond 2; 3M ESPE, St. Paul, Minnesota, United States) applied per the manufacturer′s instructions (Figures 1E and 2E).

### 3.5. Step 5—Composite Enclosure and Occlusal Verification

With the wire seated and its passive fit reconfirmed, the microhybrid resin composite (Filtek Z250; 3M ESPE, St. Paul, Minnesota, United States) encloses it, adapted with a plastic instrument or composite adapter, light‐cured per the manufacturer′s instructions, and then finished and polished to the pre‐preparation height and contour of the incisal edge, as in a Class VI restoration, so that pre‐existing occlusal contacts are preserved. Contacts are marked and verified in centric occlusion and excursions (Figures 1F–G, 2F–G and 3).

### 3.6. Step 6—Radiographic Record

A postoperative radiograph is taken where indicated, recording the seated wire and the confinement of the restoration to the prepared tunnel (Figure 1H).

## 4. Clinical Significance

The role proposed is narrow: maintaining the anterior segment through an interval that ends in extraction. That interval is not confined to implant rehabilitation—it arises equally where a removable prosthesis is intended and may then be longer. In Case 1, the form of replacement was left open at the time of splinting; the interval was the same either way.

The technique suits that role. It uses stainless‐steel wire, conventional adhesive, and composite resin, stocked in any general practice; delivery is completed in a single appointment with no laboratory stage, and the restoration adds nothing to the lingual surface for the tongue to find—a consideration of weight in elderly patients with little tolerance for intraoral change.

Its disadvantages—a visible metal element, composite wear under incisal function, and irreversible removal of tooth structure—are permanent objections in a tooth intended for retention, where a preparation‐free approach such as FRC splinting is preferred. In a tooth with an agreed extraction date, they expire with the tooth. Where the metal display is nonetheless a concern, tooth‐colored coated stainless‐steel wires are commercially available in comparable dimensions; both cases reported here used the uncoated wire.

## 5. Discussion

The splinting literature concerns teeth intended for retention [8, 15], and the distinction changes what the clinician is trying to achieve. Where the objective is periodontal management, the questions are attachment level, pocket depth, and tooth survival. Where extraction has been agreed and only timing remains open, they are narrower: will the segment stay firm, will the patient tolerate it, and will it last as long as the sequence requires. The two intervals differed in kind: In Case 2, the sequence was settled and ran to plan; in Case 1, the form of replacement and its timing both remained open.

### 5.1. Relation to FRC Splinting

FRC splinting is the contemporary reference approach for provisional stabilization, and its advantages here are real: no tooth preparation and a considerably better appearance [9, 10]. Its reported performance is mixed, with debonding and delamination predominating among failures and outcomes being sensitive to operator technique [9].

The two also differ in kind. FRC splinting is applied extracoronally, bonded to unprepared enamel; the arrangement described here is intracoronal and removes a limited amount of tooth structure. What differs beyond that is the balance of considerations, not the performance of either method. Where a tooth is to be kept, irreversible removal of structure weighs heavily and the absence of preparation is decisive. Where it will be extracted at a known endpoint, that consideration loses much of its force, whereas availability of materials and simplicity of delivery gain relevance—a difference between indications.

Lingual placement occupies the surface the tongue contacts in function and obstructs interdental floss across the splinted segment, as does a bonded splint of any design [1, 6, 11]. The incisal site exchanges these for exposure to incisal function and leaves the lingual surface untouched. Neither patient reported phonetic disturbance nor awareness of the restoration.

### 5.2. Relation to Earlier Intracoronal and Bonded‐Wire Techniques

Intracoronal splinting—a preparation cut within the clinical crown to receive a wire or ligature, then sealed with restorative material—is well described. Klassman and Zucker [12] prepared the labial, lingual, and proximal surfaces to approximately the amelodentinal junction, at the level of the contact areas, and ligated with dead soft wire in figure‐of‐eight loops. Schmid et al. [13] cut circumferential grooves 1.0–1.5 mm deep into the occlusal third, ligated with polyester yarn, and sealed with acid‐etch composite, reporting 51 splints in 34 patients at 1 year. Baar et al. [14] cut a channel 2 mm deep in the incisal edge to carry a 2‐mm wide orthodontic wire or a braided archwire, and listed six advantages, among them minimal blockage of the interdental zone, minimal disturbance of occlusion and simple execution—considerations the present rationale repeats.

The last of these is close to the arrangement described here, and the resemblance is better stated plainly than qualified. What differs is narrow. Retention here is adhesive, using selective etching and a bonding agent, whereas Baar avoided etching deliberately and relied on mechanical retention within the channel; no orthodontic phase precedes the preparation; and the endpoint is not the same. In all three reports, the splinted teeth were to be kept—semipermanently in Schmid′s series, and over the long term in Baar′s, a 5‐year follow‐up being reported as evidence of success. Here, they are agreed for extraction, and the splint ends when the sequence reaches them.

That difference also suggests why such preparations became sparse in the literature after the early 1990s. The retention they provided was later obtainable without cutting tooth structure: As enamel bonding matured and FRC came into use, the principal objection to an intracoronal preparation—irreversible loss of tooth structure, which Baar recorded as the disadvantage of his own technique—could be avoided altogether in a tooth intended for retention—and, as implant therapy matured, such teeth were increasingly extracted and replaced rather than splinted at all. That objection carries a different weight in a tooth that will be extracted, and the indication described here is one in which it does not apply.

The mechanical arrangement is also that of a fixed orthodontic retainer. Purpose, site, and periodontal context differ: positional retention of sound teeth after orthodontic treatment, against interim stabilization of teeth awaiting extraction; a lingual or cingulum surface, against a tunnel within the incisal third; intact support, against severely reduced attachment with existing attrition. The long orthodontic record of bonded wire [16, 17] is reassuring as to the interface, though it cannot be transferred to this indication.

### 5.3. Potential Complications

Neither patient experienced a complication; two patients provide no basis for estimating a rate. The expected mechanisms are fracture or permanent deformation of the wire under repeated incisal loading; wear, staining, and marginal degradation of the composite; secondary caries at the composite–tooth interface, along a margin lying within a tunnel and not readily inspected; postoperative sensitivity or pulpal injury in vital teeth, given the proximity of the preparation and the heat of cutting; and accelerated wear of opposing teeth where clearance is inadequate—the last, a reason for the case‐selection requirement in Section 3.

### 5.4. What These Cases Do and Do Not Show

What these two cases demonstrate is that the restoration remained intact and bonded for the interval required and was tolerated without complaint. The limitations are considerable. Two patients cannot support any statement about efficacy, longevity, or complication rates, and both were treated by one clinician, so results may reflect individual technique rather than reproducibility. No mobility index was recorded, so the stabilization described is a clinical impression, not a measurement; no plaque or bleeding index was taken, leaving the hygiene reasoning untested; and no periodontal charting or vitality testing was done, so nothing can be said of periodontal or pulpal consequences. In Case 2, the occlusal vertical dimension and existing contacts were unchanged, but the distribution of functional load shifted as posterior support was restored, and the mechanical behavior of the configuration remains uncharacterized. Where the sequence waits on the patient rather than on the clinician, the interval is open‐ended, and the two cases here bound it only at 5.5 and 12 months.

Prospective work would need to specify what it measures. Useful endpoints: mobility by a standardized index at baseline, after placement and at fixed intervals until extraction; patient‐reported comfort, function, speech, and awareness of the restoration through the interval; splint survival measured against a date set in advance, with time to first debonding; chair time, number of appointments, material cost and operator‐rated ease of delivery, compared directly with FRC splinting in matched indications; pulp vitality monitored in vital teeth after preparation; plaque and bleeding indices at the splinted sites, to test the hygiene rationale directly; and whether reliable interim stabilization widens the sequencing options open to the clinician. The mechanical behavior of the configuration is a separate question from clinical performance: It cannot be characterized from patient observation and would require laboratory loading of the assembly before any mechanical claim could be made.

## 6. Conclusions

In two patients undergoing staged rehabilitation, an ITTS proved technically straightforward and was well‐tolerated, remaining intact for the interval the treatment sequence required—5.5 months in one case, approximately 12 months in the other.

No conclusion regarding hygiene, comfort, or periodontal outcome can be drawn from uncontrolled observation of two patients. It is offered as one splinting option for the pre‐extraction interval of staged rehabilitation, assembled from materials present in any general practice. Section 5.4 sets out what would need to be measured to establish whether it holds any advantage over established alternatives.

## Author Contributions

**Fahad Hussain Al-Qahtani:** conceptualization, methodology, investigation, resources, data curation, writing—original draft, writing – review and editing, visualization, project administration.

## Funding

No funding was received for this manuscript.

## Disclosure

The author has read and approved the final version of the manuscript. The corresponding author, who is also the manuscript guarantor, had full access to all of the data in this study and takes complete responsibility for the integrity of the data and the accuracy of the data analysis.

## Ethics Statement

Both procedures were carried out as part of routine clinical care, using established materials and instruments, with no experimental intervention, randomization, or departure from standard practice. Under the governing institutional policy, retrospective reporting of anonymized routine care accompanied by written patient consent does not require formal ethics committee review. Written informed consent for treatment and for publication of clinical data, photographs, and radiographs was obtained from both patients, in accordance with the Declaration of Helsinki.

## Consent

Written informed consent for treatment and for publication of clinical details and accompanying images was obtained from both patients before any intervention. Signed forms are provided as supporting material marked not for review.

## Conflicts of Interest

The author declares no conflicts of interest.

## Data Availability

The author confirms that the data supporting the findings of this study are available within the article. Identifiable clinical records and signed consent forms cannot be shared publicly in order to protect patient confidentiality.
